# RECRUITMENT AND RETENTION OF DEMENTIA CAREGIVERS: A REVIEW OF TWO STUDIES

**DOI:** 10.1093/geroni/igz038.1283

**Published:** 2019-11-08

**Authors:** Lauren Parker, Laura N Gitlin

**Affiliations:** 1 Johns Hopkins Bloomberg School of Public Health, Baltimore, Maryland, United States; 2 College of Nursing and Health Professions, Drexel University, Philadelphia, Pennsylvania, United States

## Abstract

Two of the goals of the National Strategy for Recruitment and Participation in Alzheimer’s Disease and Related Dementias Clinical Research are to “increase awareness and engagement (Goal 1)” and to “engage local communities and support participants (Goal 3)”. Leveraging the findings from two National Institute on Aging funded dementia-caregiving related research studies, ADS-Plus and The Providing Evidence-Based Approaches to Caregiver Stress, the goal of this presentation is to discuss methodologies and approaches to develop culturally competent content for outreach. Recruitment strategies from the two studies will explore the impact of race/ethnicity on recruiting Hispanic (ADS-Plus), and Black/African American populations. Further, the effectiveness of recruitment strategies from both studies will be discussed, as an effort to (1) conceptualize best practices necessary to develop and sustain equitable and sustainable community partnership, and (2) create and improve evidence-based recruitment resources.

